# Global biogeography and projection of antimicrobial toxin genes

**DOI:** 10.1186/s40168-025-02038-5

**Published:** 2025-02-04

**Authors:** Ya Liu, Yu Geng, Yiru Jiang, Jingyu Sun, Peng Li, Yue-zhong Li, Zheng Zhang

**Affiliations:** 1 State Key Laboratory of Microbial Technology, Institute of Microbial Technology, Shandong University, Qingdao, 266237 China; 2 Qilu Hospital (Qingdao), Cheeloo College of Medicine, Shandong University, Qingdao, 266035 China; 3 Suzhou Research Institute, Shandong University, Suzhou, 215123 China

**Keywords:** Antimicrobial toxins, Antagonism, Global biogeography, Anthropogenic factors

## Abstract

**Background:**

Antimicrobial toxin genes (ATGs) encode potent antimicrobial weapons in nature that rival antibiotics, significantly impacting microbial survival and offering potential benefits for human health. However, the drivers of their global diversity and biogeography remain unknown.

**Results:**

Here, we identified 4400 ATG clusters from 149 families by correlating 10,000 samples worldwide with over 200,000 microbial genome data. We demonstrated that global microbial communities universally encode complex and diverse ATGs, with widespread differences across various habitats. Most ATG clusters were rare within habitats but were shared among habitats. Compared with those in animal-associated habitats, ATG clusters in human-associated habitats exhibit greater diversity and a greater proportion of sharing with natural habitats. We generated a global atlas of ATG distribution, identifying anthropogenic factors as crucial in explaining ATG diversity hotspots.

**Conclusions:**

Our study provides baseline information on the global distribution of antimicrobial toxins by combining community samples, genome sequences, and environmental constraints. Our results highlight the natural environment as a reservoir of antimicrobial toxins, advance the understanding of the global distribution of these antimicrobial weapons, and aid their application in clinical, agricultural, and industrial fields.

Video Abstract

**Supplementary Information:**

The online version contains supplementary material available at 10.1186/s40168-025-02038-5.

## Background

Microorganisms exert influence across all aspects of human life, shaping ecosystems, agriculture, industrial processes, health, and disease management, among other fields. Whether in natural environments or within the human body, microbial communities generally exhibit high levels of diversity and density. To vie for scarce resources and niches, microorganisms employ various strategies to inhibit the growth of their competitors, with the most prevalent strategies being the production of antibiotics and the secretion of antimicrobial toxin proteins [1, 2]. Increasing evidence suggests the central importance of antagonism in microbial life, underscoring the significance of research into antagonistic mechanisms for understanding and manipulating essential microbial communities [3, 4]. However, despite the existence of genomic and laboratory evidence unequivocally indicating the widespread prevalence of microbial antagonism mediated by antimicrobial toxins, the focus on antimicrobial toxins has only recently begun to intensify compared with that on antibiotics [5, 6].

Although only a limited number of antimicrobial toxins have been experimentally verified, they exhibit diverse sequence compositions, activities, and delivery mechanisms [7]. Antimicrobial toxins can be broadly classified into (i) diffusible molecules that function in a contact-independent manner, such as bacteriocins, which can be directly released into the extracellular milieu and (ii) antimicrobial proteins that rely on protein secretion apparatuses to facilitate their contact-dependent delivery to target cells [5, 8, 9]. These secretion apparatuses include type IV, type V, type VI, and type VII secretion systems as well as extracellular contractile injection systems (eCISs) and outer membrane exchange systems. To effectively inhibit the growth of competitors, antimicrobial toxins often possess enzymatic activity, requiring only minute quantities to disrupt critical molecular structures in target cells. These include nucleases targeting DNA or RNA, phospholipases or pore-forming proteins targeting the cell membrane, glycoside hydrolases or proteases targeting the cell wall, NADases disrupting the cellular energy balance, and ADP-ribosyltransferases targeting bacterial tubulin-like proteins to interrupt the division of competing cells, among others [10–12]. Many antimicrobial toxin proteins exhibit a modular nature, resulting in extraordinary sequence diversification through recombination of different N-terminal delivery or marker domains with C-terminal toxin domains [13–17].

Many antimicrobial toxins have been demonstrated to be involved in the pathogenesis of pathogens in humans, animals, and plants [5]. Antimicrobial toxins produced by important human pathogens, such as *Vibrio cholerae*, *Yersinia pestis*, *Acinetobacter baumannii*, and *Pseudomonas aeruginosa*, as well as pathogens significantly harmful to animals, plants, crops, and aquaculture (e.g., *Xanthomonas citri*, *Pseudomonas syringae*, *Vibrio parahaemolyticus*, and *Vibrio alginolyticus*), can directly target eukaryotic cells, disrupting cellular functions and facilitating pathogen infection [8, 11]. In addition to directly affecting eukaryotic hosts, antimicrobial toxin-mediated antagonism plays a dual role in the resistance of human-associated microbial communities to pathogen invasion and colonization. On the one hand, symbiotic bacteria of the host can resist the invasion of pathogens by secreting antimicrobial toxins. On the other hand, some pathogens utilize antimicrobial toxins to compete with indigenous symbiotic bacteria, thereby invading ecosystems and causing disease [18–21]. More importantly, strong experimental evidence in recent years indicates that antimicrobial toxins initiate cancer through mediating mutagenesis [22]; for example, colibactin, cytolethal distending toxin, and *Bacteroides fragilis* toxin cause mutational signatures found in colorectal, head and neck, and urinary tract cancers [23–26].

Microbial resistance to antibiotics has become one of the most severe threats to public health and food safety, necessitating the development of new countermeasures. As antimicrobial weapons in nature rivalling antibiotics, antimicrobial toxins provide a potential solution [27]. Antimicrobial toxins can target the treatment of specific antibiotic-resistant bacterial infections through strategies such as the use of bacteriophages, bacteriocins, or engineered secretion systems. Efforts have been made to engineer the type VI secretion system (T6SS) as a vehicle for delivering customizable antimicrobial toxins [28, 29]. Recent research has demonstrated the precise targeted delivery of antimicrobial toxins into human cells and mouse brains via engineered eCIS [30]. Antimicrobial toxins are also highly important for those seeking to manipulate and engineer microbial communities for human benefit. Microbial interactions mediated by antimicrobial toxins are crucial for the robustness, scalability, and programmability of artificial communities [31–33]. In addition, the enzymatic activity of antimicrobial toxins has the potential to lead to new biotechnological applications, such as DNA base editing [34].

Although increasing information highlights the critical role of antimicrobial toxins in ecosystems and human health [5, 10, 35], a comprehensive assessment of the distribution of antimicrobial toxin genes (ATGs) in the global biosphere has not been conducted. Here, by correlating 10,000 samples from the Earth Microbiome Project (EMP) with over 200,000 microbial genome datasets, we identified 4400 ATG clusters. This allowed us to comprehensively evaluate the abundance and diversity of ATGs in microbial communities across various habitats and to construct and analyze a global atlas of ATG distribution along with the drivers.

## Methods

### Microbial community genetic information collection

The EMP is a large-scale collaborative initiative aimed at understanding the ecological patterns of microbial communities and habitats on Earth [36]. The analysis utilized a subset of 10,000 samples released by EMP, which were carefully selected to represent a range of habitat types and research relevance. A total of 262,011 amplicon sequence variants (ASVs) and their abundance and nucleic acid sequence information were collected from these 10,000 samples using the Deblur software [37].

The NCBI Reference Sequence database is a collection of selected nonredundant sequences representing complete or framework genomes [38]. We obtained a dataset encompassing 217,614 bacterial or archaeal genomes. Mapping the 262,011 ASV sequence data from EMP to the sequenced genome information of these 217,614 genomes enabled the assessment of the proportions of genome-sequenced cells and taxa in microbial communities. The 16S rRNA gene (V4 region) sequences were mapped with a 100% identity threshold to better differentiate closely related organisms. Under this premise, for the 10,000 EMP samples studied, the median proportions of cells and taxa with known genomic information reached 40.2% (20.5–88.1%) and 21.3% (11.4–57.0%), respectively. Notably, the sequenced proportion of the microbial genome in biomes has further increased compared with our previous evaluation [39].

### Identification of ATGs

Based on the characteristics of ATGs, ATGs were identified in the 6019 mapped genomes. ATGs are secreted extracellularly to exert their antagonistic interaction, either as diffusible molecules (such as bacteriocins) or as effectors delivered directly to target cells through various secretion systems. A notable characteristic of ATGs is the fusion of specific N-terminal secretion-related marker domains with different toxic domains. Another remarkable characteristic is that the loci encoding ATGs also encode immunity proteins that neutralize their toxicity. Additionally, some T6SS-associated ATGs can be identified through adjacent adaptor protein genes because adaptor proteins are mediators that help to load their cognate effectors onto the T6SS spike complex. ATGs associated with outer membrane exchange systems are secreted via signal peptides.

On the basis of our previously constructed prokaryotic antimicrobial toxin database (PAT database) [7], sequence information for ATG proteins, immunity proteins, secretion-related markers, and adaptors was collected. Secretion-related markers include trafficking domains, repeat domains, pre-toxins, and conserved motifs [9, 10, 13]. Trafficking domains include, for example, VgrG [40], PAAR [41, 42], LXG [43], DUF4157 [44], WXG100 [45], SpvB [13], TANFOR [46], Phage_Mu_F [13], and FhaB [47]. Repeat domains include Haemagg_act [48] and RHS repeat [49]. Pre-toxins include PT-HINT [16], PT-TG [16], and PT-VENN [50]. Conserved motifs include Mix [51] and Fix [52]. The DUF4123 [53], DUF2169 [54], DUF1795 [55], and PRK06147 [56] protein families were described as adaptors. In total, we identified 149 ATG families, 73 immunity protein families, 42 secretion-related marker families, and 4 adaptor families (Supplementary Data 1).

**Fig. 1:**
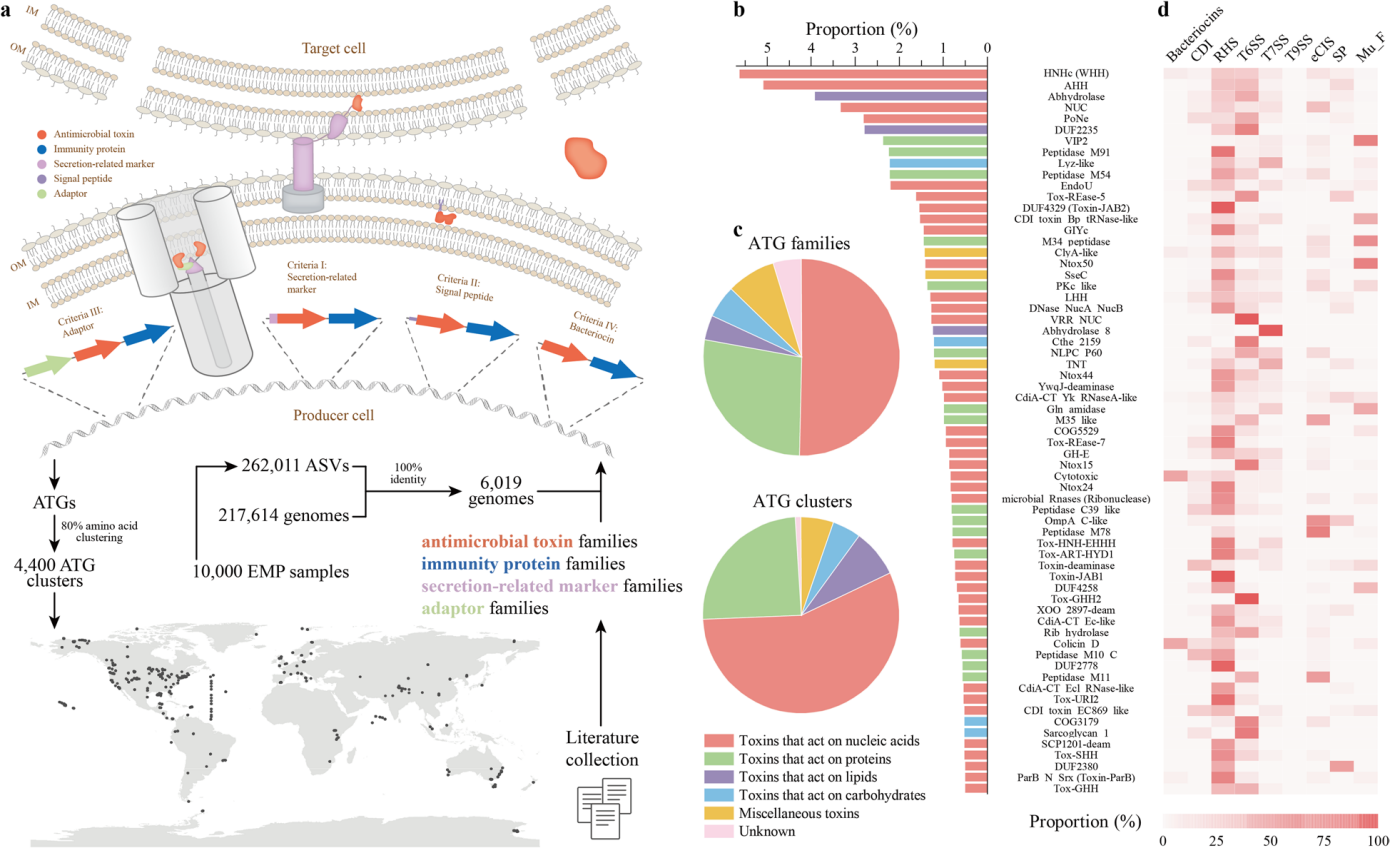
Diverse antimicrobial toxins enhance the antagonistic arsenal of microbial communities. **a** Workflow for the identification of antimicrobial toxin genes (ATGs) in microbial communities. Amplicon sequence variants (ASVs) from the Earth Microbiome Project (EMP) samples were mapped to sequenced genomes based on 100% 16S rRNA gene sequence identity and then scanned for ATGs in the obtained genomes using a series of known antimicrobial toxin features, such as secretion-related marker domains, signal peptides, and adaptors. The identified ATGs were clustered into clusters (Methods). **b** Identified ATGs from 149 families. Only the top 65 families, each comprising more than 0.5% of the total, are displayed. **c** Classification of potential targets of ATGs. Targets were determined based on known functional members within each family, and 149 families and 4400 clusters were statistically analyzed respectively. Red represents toxins that act on nucleic acids, green represents toxins that act on proteins, purple represents toxins that act on lipids, orange represents toxins with other functions, and pink represents toxins with unknown functions. **d** Interconnected associations between ATG families and potential delivery modes. Delivery modes are predicted based on secretion-related marker domains. The heatmap color indicates the percentage of antimicrobial toxins associated with each delivery mode within the family

Using RPS-BLAST (with an *E*-value threshold of 0.01), we scanned all genes encoding the aforementioned ATG families, immunity proteins, secretion-related markers, and adaptors in the 6019 sequenced prokaryotic genomes included in the EMP samples [57]. On the basis of this analysis, we further examined the domain architectures and gene neighborhoods of the ATG families (candidate ATGs) (Fig. 1). A gene was determined to encode an ATG if it met any of the following four criteria: (i) the N-terminus of the protein product encoded by the gene contained at least one secretion-related marker domain; (ii) the downstream neighborhood of the gene encoded a corresponding immunity protein, with the N-terminus encoding a signal peptide; (iii) in addition to the downstream neighborhood encoding the corresponding immunity protein, the upstream neighboring gene also encoded an adaptor; and (iv) the gene encoded a bacteriocin family, and its downstream neighborhood encoded the corresponding immunity protein. Ultimately, a total of 4774 ATGs were identified (Supplementary Data 1). Notably, these identified ATGs exhibited secretion characteristics associated with specific delivery modes, making them unlikely to be confused with toxin-antitoxin systems. Toxins in toxin-antitoxin systems typically cause programmed cell death of the host cell and are not normally secreted extracellularly.

### Calculation of ATG diversity and abundance in communities

Using the ATGs identified from the community genetic information, we evaluated the global distribution patterns of ATGs in terms of diversity and abundance (Supplementary Data 2). The ATGs we identified belong to 149 protein families and were clustered based on amino acid sequences using CD-HIT (with a minimum identity threshold of 80% and a minimum sequence coverage of 80%) [58], resulting in 4400 unique homology clusters. The ATG diversity in the community was calculated based on the number of families and homology clusters to which these ATGs belonged, respectively. The relative abundance of ATGs in each community was calculated based on the number of ATGs and 16S rRNA genes encoded by the mapped genomes in the community, representing the copy number of ATGs normalized against the cell number (genes/cell):

$$\text{ATG abundance}=\frac{{\sum }_{i=1}^{n}{N}_{i{\text{ATG}}}\times {R}_{i}/{N}_{i{\text{16S}}}}{{\sum }_{j=1}^{m}{R}_{j}/{N}_{j{\text{16S}}}}$$

Here, *m* is the total number of ASVs mapped to the genomes in the community, *n* is the total number of ASVs with genome-encoded ATGs, *i* and *j* represent specific ASVs in the community, ${R}_{i}$ represents the relative abundance of the ASV, ${N}_{i{\text{ATG}}}$ represents the number of ATGs in the genome, and ${N}_{i{\text{16S}}}$ represents the number of 16S rRNA genes in the genome.

To investigate local abundance patterns, the abundance distribution of samples in a community was described using a log-normal model to assess the shape of the ATG cluster abundance distribution in each of the 10,000 samples. In spatial occupancy studies, occupancy frequency distributions described the spatial distribution of biological units across a set of communities and classify these units along a distribution gradient from spatially restricted to ubiquitous. The occupancy of ATGs within each habitat was estimated by calculating the number of samples in which an ATG was detected and dividing it by the total number of samples from that habitat (ranging from 0 to 1).

The EMP ontology (EMPO) is used to classify samples into different habitats within a hierarchical framework, capturing the primary dimensions of microbial community diversity. This classification system identified 17 distinct microbial habitats at 3 levels from 10,000 samples. These habitats are further divided into two broader categories: free-living or host-associated at level 1 and saline or non-saline (for free-living) or animal or plant (for host-associated) at level 2 [36]. The hypersaline (saline) habitat was not analyzed because only 13 samples were included. The remaining 16 habitats contained between 81 samples (aerosol, non-saline) and 987 samples (animal surface). Human-associated communities, a subset of host-associated communities, are derived from 4 habitats: the gut (216 samples), nasal/pharyngeal (253 samples), oral (447 samples), and skin (346 samples) habitats.

### Machine learning

We selected 97 spatial covariates (Supplementary Data 3), including longitude and latitude, as features to predict the global distribution of ATG diversity and abundance [59–67]. All 97 variables can be categorized into 8 groups: anthropogenic factors, temperature, soil properties, radiation, precipitation, moisture, other climatic variables, and land use and others. To obtain atlases with uniform resolution, we resampled all datasets using the nearest neighbor method to match the same resolution.

First, we processed the EMP data by merging samples with identical coordinates and calculating the average diversity and abundance. Then, we obtained the 97 spatial covariates corresponding to the sample locations through ArcGIS Pro. The samples were split into a training set and a testing set at an 8:2 ratio for machine learning.

We used the training set to predict ATG diversity and abundance through the random forest algorithm. Initially, we employed the recursive feature elimination algorithm to identify the optimal feature set. Next, we used a grid search to optimize the model’s hyperparameters, achieving the best hyperparameter combination. Both steps were based on tenfold cross-validation to reduce overfitting. The training set was randomly divided into 10 equal subsets, with 9 subsets used for training the model and the remaining subset used for model evaluation. This process was repeated for 10 rounds, and the resulting tenfold cross-validation *R*^2^ was used to assess model performance. The final tenfold cross-validation *R*^2^ on the training set for the model predicting ATG cluster diversity was 0.691, that for predicting family diversity was 0.679, and that for predicting toxin abundance was 0.577. We validated our models on the testing set, with all three models achieving *R*^2^ values exceeding 0.5, indicating that our models also performed well when dealing with untrained data.

Finally, we set 10 different random seeds to train 10 independent random forest models, calculated the average of the 10 predicted values as the final prediction, and computed the coefficient of variation of the 10 predicted values to assess model uncertainty.

The importance of each variable was also determined through machine learning to evaluate the most critical drivers affecting ATG diversity and abundance. We used the variable importance tool from the caret package in R, employing permutation variable importance measures to assess the relative importance of all selected variables. This tool used out-of-bag estimates to calculate the mean squared error for each regression tree. To better compare variable importance, we standardized the importance of these variables within a range of 0 to 100%, obtaining their relative importance (Supplementary Data 4, 5, and 6) [68].

### Statistical analysis

Data analysis was primarily conducted using R (version 4.3.1) and related packages. The caret and randomForest packages were employed for recursive feature elimination, hyperparameter tuning, and calculating the relative importance of variables. The ggplot2 package was used to generate some of the figures. ArcGIS Pro was utilized to extract spatial covariates corresponding to sample locations and map the global distribution of ATG diversity and abundance, and Origin was used for generating other figures.

Nonlinear models were used to determine occupancy and abundance distribution trends. A simple linear model was applied to fit the relationship between ATG cluster abundance and occupancy. For all comparisons, the Wilcoxon signed-rank test was used to compare differences between paired samples, whereas the Wilcoxon rank-sum test was used to compare differences between independent samples. Univariate associations were determined using Spearman’s rank correlation.

## Results

### Diverse antimicrobial toxins enrich the antagonistic arsenal of microbial communities

We determined the composition of antimicrobial toxins for the ASVs contained in 10,000 samples of the EMP dataset (Fig. 1a). To accomplish this goal, we initially mapped the 16S rRNA gene sequences to microbial genomes in the NCBI reference sequence database with 100% sequence identity (“Methods” and Supplementary Fig. S1). We subsequently scanned the obtained 6019 genomes for ATGs using a series of known antimicrobial toxin features.

We identified a total of 4774 ATGs (Supplementary Data 1). These ATGs originate from as many as 149 protein families, with only 2 families comprising more than 5% (WHH, 5.6%; AHH, 5.1%), whereas 65 families exceed the 0.5% (Fig. 1b). The application of stricter criteria requiring antimicrobial toxins to possess at least 80% amino acid sequence identity and coverage yielded 4400 unique homologous protein clusters, with more than 95% of the clusters containing only 1 member. Notably, 56.1% of these ATG clusters are present in human-associated habitats (1262 samples), including the gut, nasal/pharyngeal, oral, and skin, covering 140 out of the 149 ATG families. Thus, antimicrobial toxins in microbial communities are extremely diverse in sequence, especially in human-associated communities.

The targets and activities of antimicrobial toxins are also diverse, with nucleic acids being the most common potential targets (Fig. 1c). More than half of the ATG families (75) and clusters (2481) are associated with nucleic acids, followed by proteins, carbohydrates, lipids, and some with miscellaneous or unknown functions. Based on delivery or marker domains, we predicted potential delivery mechanisms for antimicrobial toxins (Fig. 1d). We identified 10 delivery modes, capable of delivering antimicrobial toxins from 5 (T9SS) to 126 (RHS) families. Surprisingly, 50 ATG families can be delivered by at least 5 different modes, and more than 80% of the families (122) are associated with at least 2 delivery modes. Thus, complex and diverse associations exist between ATG families and delivery mechanisms, significantly enriching the arsenal of microbial antagonism.

### Distribution of ATGs exhibits widespread differences across diverse habitats

**Fig. 2:**
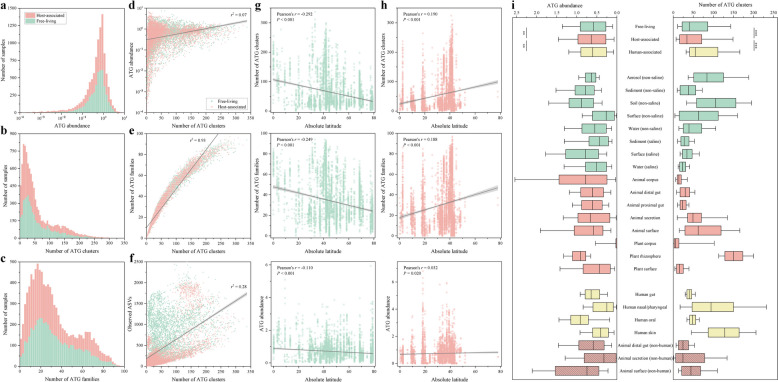
Widespread habitat differences in ATG distribution. **a** Sample number statistics of different ATG abundances in the community based on 10,000 EMP samples. **b** Statistics on the number of samples with different ATG cluster numbers in the community. **c** Sample number statistics of different ATG family numbers in the community. **d** Weak correlation between the diversity and abundance of ATG clusters in communities. **e** Strong positive correlation between the diversity of ATG clusters and the number of families in communities. **f** Positive correlation between the diversity of ATG clusters and biodiversity in communities. Community biodiversity is represented by the number of observed ASVs. **g**, **h** Latitudinal distribution of ATG cluster diversity, family diversity, and abundance in free-living (**g**) and host-associated (**h**) communities. For all the scatter plots, the lines indicate the best linear fit, the shaded areas represent the 95% confidence intervals, and *r*.^2^ denotes the coefficient of determination. Pearson’s correlation tests were used to examine the correlations between absolute latitude and ATG cluster diversity, family diversity and abundance, and the *P*-value indicated statistical significance. **i** Statistics of ATG abundance and cluster diversity in various habitats. Habitat classification is based on EMPO, where 10,000 samples are initially divided into free-living (green) and host-associated (red) categories and further subdivided into 17 types of habitats. The hypersaline habitat is not shown due to the small number of samples (13), whereas the other 16 habitats range from 81 (aerosol (non-saline)) to 987 (animal surface) samples. The human-associated habitat (yellow) is a subset of the host-associated habitat, including the gut (216 samples), nasal/pharyngeal (253 samples), oral (447 samples), and skin (346 samples) habitats. For the box plots, the middle line represents the median, the box indicates the 25th–75th percentiles, and the error bars represent the 10th–90th percentiles of the observations. Comparisons between bins were conducted using the Wilcoxon rank-sum test, **P < 0.01, ***P < 0.001

Based on 10,000 EMP samples, we calculated the composition of ATG in each community (Supplementary Data 2). Nearly all of these microbial communities (99.1%) were found to harbor detectable ATGs, with a median ATG abundance of 0.61 (0.27–0.94) genes/cell (Fig. 2a). Each community encoded a median of 37 (18–80) ATG clusters and up to 26 (15–45) ATG families (Fig. 2b, c). The number of ATG clusters encoded in a community was strongly correlated with the number of families (*r*^2^ = 0.93) but weakly correlated with ATG abundance (*r*^2^ = 0.07) (Fig. 2d, e). Although the total number of ATG clusters (4400) was nearly 30 times greater than the number of ATG families (149), the average number within each community was approximately 1.5 times greater. ATGs from the same family in different communities often belong to different clusters. Importantly, the number of ATG clusters encoded in a community was positively correlated with biodiversity (*r*^2^ = 0.28), indicating that communities with greater biodiversity also exhibited greater ATG diversity (Fig. 2f and Supplementary Fig. S2). These results suggest that microbial communities worldwide commonly encode complex and diverse antimicrobial toxins.

Based on the EMPO environmental classification, ATGs in the free-living communities exhibited a latitudinal diversity gradient (LDG), with cluster diversity (Pearson’s *r* = − 0.292, *P* < 0.001), family diversity (Pearson’s *r* = − 0.249, *P* < 0.001), and abundance (Pearson’s *r* = − 0.110, *P* < 0.001) all showing weak but significant negative correlations with absolute latitude (Fig. 2g). Contrary to the typical LDG, host-associated microbial communities displayed a positive correlation with absolute latitude (cluster diversity: Pearson’s *r* = 0.190, *P* < 0.001; family diversity: Pearson’s *r* = 0.188, *P* < 0.001; abundance: Pearson’s *r* = 0.032, *P* = 0.020) (Fig. 2h). Comparatively speaking, the abundance of ATGs encoded in free-living communities was significantly lower than that in host-associated communities (0.58 versus 0.64, Wilcoxon rank-sum test, *P* = 0.002). However, cluster diversity (40 versus 34, Wilcoxon rank-sum test, *P* < 0.001) and family diversity (28 versus 24, Wilcoxon rank-sum test, *P* < 0.001) were significantly greater in free-living communities than in host-associated communities (Fig. 2i and Supplementary Fig. S3). Specifically, the diversity of ATGs in saline habitats was lower than that in corresponding nonsaline habitats, whereas plant-related habitats (excluding plant rhizosphere similar to soil) presented lower diversity than did animal-related habitats. In addition, the cluster diversity, family diversity, and abundance of ATGs were reduced under extreme environments (e.g., hypersaline, high/low temperature, or high/low pH) (Supplementary Fig. S4).

Human-associated communities are subsets of host-associated communities. However, in comparison with the overall host-associated communities, the human-associated communities showed a slightly lower ATG abundance (0.60 versus 0.64, Wilcoxon rank-sum test, *P* = 0.001). Nonetheless, both cluster diversity (55 versus 34, Wilcoxon rank-sum test, *P* < 0.001) and family diversity (35 versus 24, Wilcoxon rank-sum test, *P* < 0.001) were significantly greater (Fig. 2i and Supplementary Fig. S3). In terms of ATG cluster diversity, each human-associated habitat significantly surpassed its corresponding animal (nonhuman) habitat. Specifically, the human gut habitat exceeded the animal distal gut (nonhuman) habitat (40 versus 24, Wilcoxon rank-sum test, *P* < 0.001), the human nasal/pharyngeal (94 versus 24, Wilcoxon rank-sum test, *P* < 0.001) and oral (48 versus 24, Wilcoxon rank-sum test, *P* < 0.001) habitats exceeded the animal secretion (nonhuman) habitat, and the human skin habitat exceeded the animal surface (nonhuman) habitat (128 versus 44, Wilcoxon rank-sum test, *P* < 0.001). Among the four human-associated habitats, the gut and oral habitats had greater ATG abundance than did the nasal/pharyngeal and skin habitats, but their cluster diversity was much lower than that of the other two habitats.

### Most ATG clusters are rare but not habitat specific

**Fig. 3:**
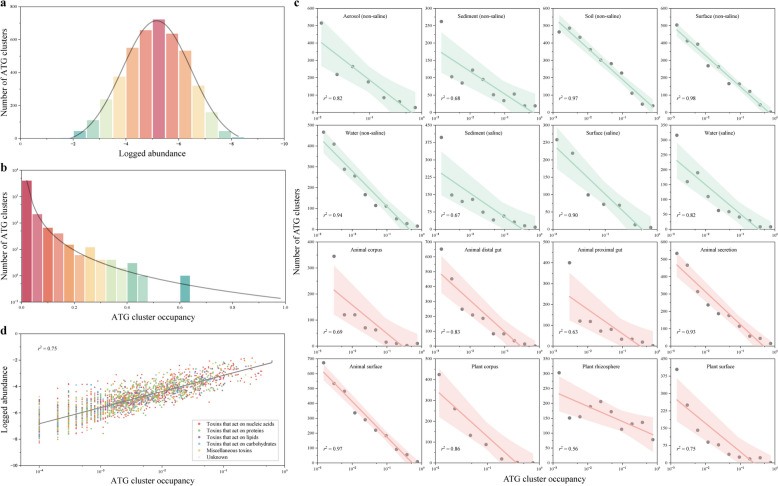
Majority ATG clusters are rare. **a** Distribution of ATG cluster abundance following a lognormal model. **b** Occupancy distribution of ATG clusters exhibiting a left-skewed unimodal pattern. The gray line indicates the best Gaussian model fit. **c** The number of ATG clusters decreased exponentially with increasing occupancy in 16 habitats. Red represents host-associated habitats, and green represents free-living habitats. **d** A positive correlation between ATG cluster occupancy and abundance on a global scale. ATG clusters with different activities of action are represented by different colors. Lines indicate the best linear fit, shaded areas represent the 95% confidence intervals, and *r*^2^ denotes the coefficient of determination

Taking the cluster as a unit, we employed a macroecological model to investigate the distribution patterns of ATGs. Across global and various habitat types, the relative abundance distribution of ATG clusters conformed to a log-normal model, with moderate abundance being the most prevalent (Fig. 3a and Supplementary Fig. S5). Furthermore, the occupancy distribution of the ATG cluster exhibited an obvious unimodal pattern, with as many as 73.9% of the clusters appearing in less than 1% of the communities (Fig. 3b). In each habitat category, the occupancy distribution was highly left mode, with the number of clusters exponentially decreasing as the occupancy increased (Fig. 3c). Whether observed globally or within specific habitats, the majority of ATG clusters were rare, occurring only in select communities. The abundance of ATG clusters showed a strong positive correlation with occupancy, adhering strictly to a distribution pattern from low-abundance and narrow-range rare types to high-abundance and wide-range common types, regardless of their functional activities (Fig. 3d and Supplementary Fig. S6).

**Fig. 4:**
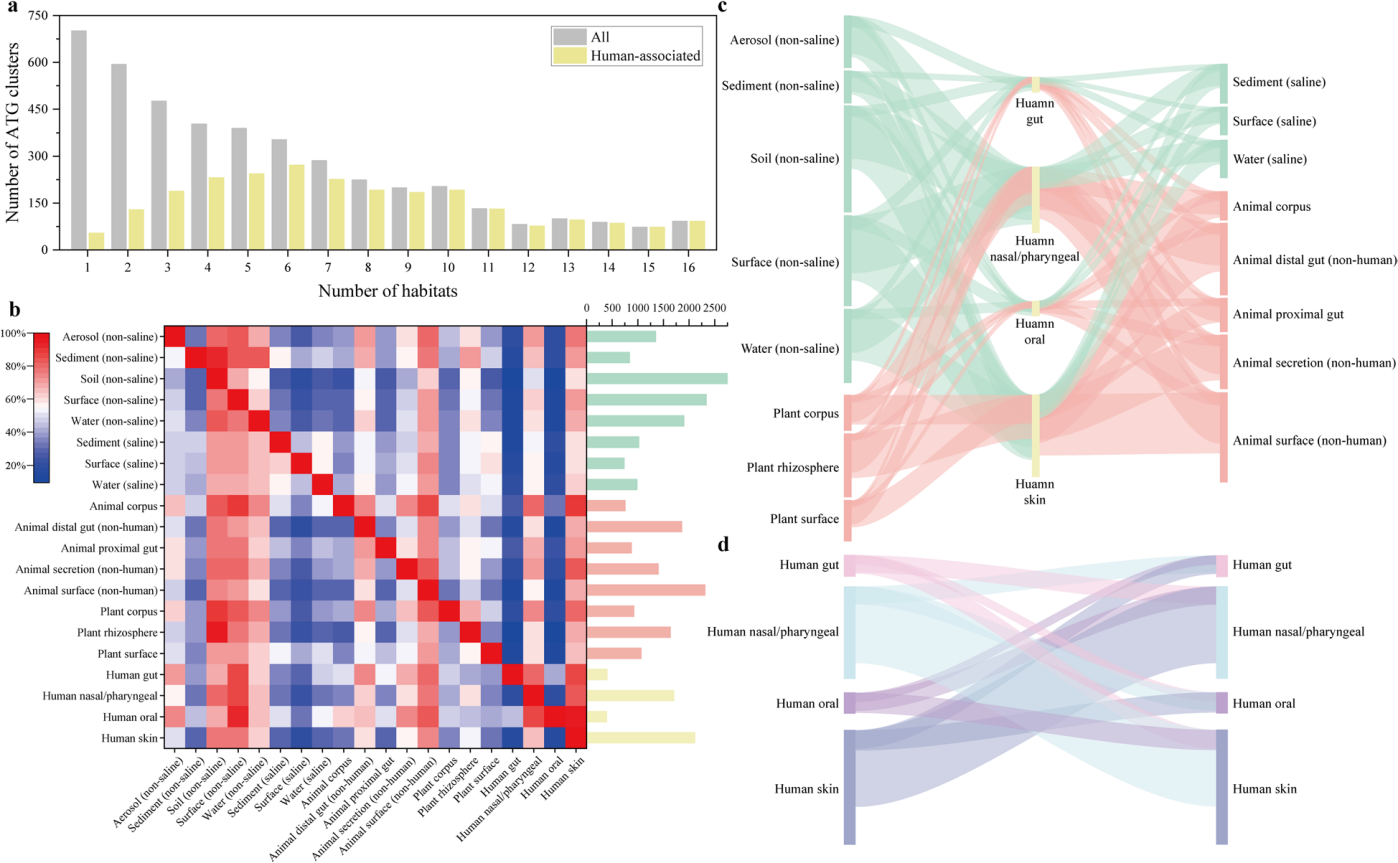
Most ATG clusters are shared among habitats. **a** Statistical analysis of the number of ATG clusters across habitats. ATG clusters occurring in human-associated habitats are indicated in yellow. 16 EMPO_3 habitats were used. **b** Proportion of shared ATG clusters between pairs of habitats. The right bar graph shows the total number of ATG clusters occurring in each habitat (as the denominator). The colors in the figure represent the percentage of shared ATG clusters between habitats, ranging from blue to red. **c** Extensive sharing of ATG clusters between human-associated habitats and other habitats. The width of each band represents the number of shared ATG clusters. Red represents host-associated habitats, green represents free-living habitats, and yellow represents human-associated habitats. **d** Sharing of ATG clusters among four human-associated habitats

In stark contrast to their rarity, as many as 84.1% of the ATG clusters were shared among habitats (Fig. 4a). Given that the vast majority of ATG clusters contain only a single member, their widespread distribution across habitats can be attributed primarily to the ability of ASVs carrying ATGs to survive in diverse habitats. Notably, ATG clusters in human-associated habitats were more likely (97.8%) to be shared across different habitats. Among the ATG clusters present in at least 5 habitats, 83.9% were also found in human-associated habitats, and this percentage increased to 96.9% for the clusters present in at least 10 habitats. The sharing of ATGs between habitats was extensive, with up to 50.4% (35.7–68.9%) of the ATG clusters being shared between pairs of the 16 EMPO_3 habitats (Fig. 4b). The highest level of sharing was observed in the animal corpus habitat, where 95.6% of the ATG clusters were shared with the animal surface habitat, whereas the lowest level of sharing occurred in the soil habitat (non-saline), with 18.8% of the ATG clusters shared with the surface habitat (saline).

ATG clusters exhibit extensive sharing between human-associated habitats and other habitats (Fig. 4b, c). In terms of the proportion of ATG clusters shared with the other 15 EMPO_3 habitats, the sharing level in the human gut habitat was significantly greater than that in the animal gut habitat (51.2% versus 35.7%, Wilcoxon signed-rank test, *P* = 0.002). Similarly, human nasal/pharyngeal habitats (40.7% versus 36.3%, Wilcoxon signed-rank test, *P* < 0.001) and oral habitats (53.3% versus 36.3%, Wilcoxon signed-rank test, *P* = 0.001) displayed significantly greater sharing rates than animal secretion habitats did, and human skin habitats also significantly exceeded animal surface habitats (35.6% versus 29.6%, Wilcoxon signed-rank test, *P* < 0.001). The habitat that was most shared with human-associated habitats was the animal surface, whereas the least shared habitat was the surface (saline). Among the four human-associated habitats, the human skin habitat, which possessed the most diverse ATG clusters, covered 85.1% of the gut habitat ATG clusters, 82.8% of the nasal/pharyngeal habitat ATG clusters, and 94.9% of the oral habitat clusters (Fig. 4d).

### Mapping the global distribution of ATGs

To predict the global distribution of ATGs, we selected 95 spatial covariates along with longitude and latitude as the features for prediction (Supplementary Data 3). Using processed EMP samples, we employed the random forest algorithm and conducted feature selection and hyperparameter tuning to optimize the model based on tenfold cross-validation. Ultimately, we identified 57 covariates for predicting ATG cluster diversity, 87 covariates for predicting abundance, and 37 covariates for predicting family diversity (Supplementary Fig. S7). Additionally, we applied the coefficient of variation to quantify the uncertainty of our estimates. Despite the relatively high uncertainty in some regions (Supplementary Fig. S8), our models showed robust performance (Supplementary Fig. S9).

**Fig. 5:**
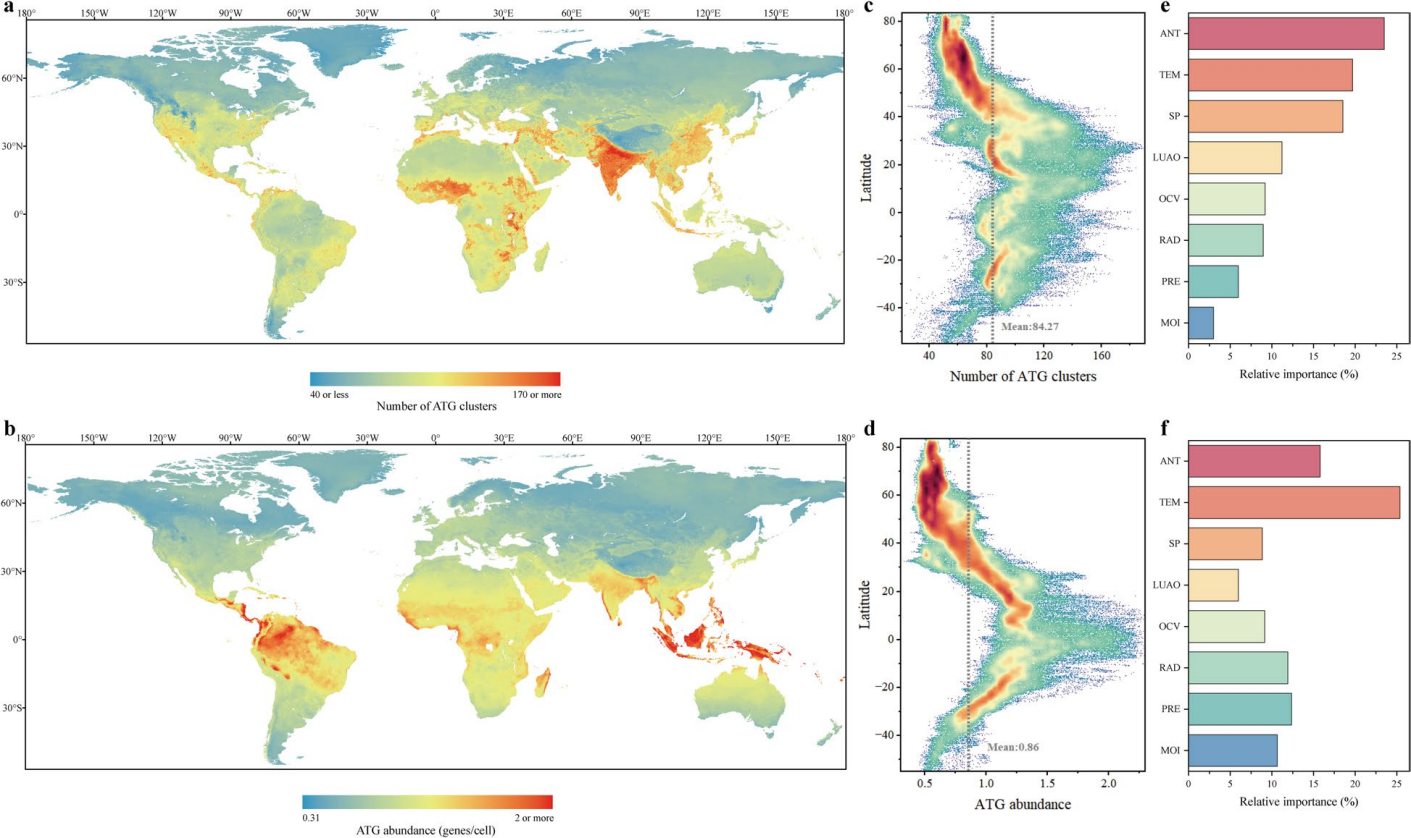
Global biogeographic patterns of ATGs. **a**, **b** Global distribution maps of ATG cluster diversity (**a**) and abundance (**b**). Global ATG cluster diversity and abundance were predicted based on a random forest model using 97 spatial covariates. Four-fifths of the samples were used as the training set, while one-fifth served as the testing set (for cluster diversity, training set tenfold cross-validation *R*^2^ = 0.691, testing set *R*^2^ = 0.587; for abundance, training set tenfold cross-validation *R*^2^ = 0.577, testing set *R*^2^ = 0.501; Supplementary Fig. S9). **c**, **d** Latitudinal distribution of global ATG cluster diversity (**c**) and abundance (**d**). The dashed lines represent the average of global ATG cluster diversity and abundance. **e**, **f** Relative importance of each category of variables for predicting ATG cluster diversity (**e**) and abundance (**f**). ANT, anthropogenic; TEM, temperature; SP, soil properties; LUAO, land use and others; OCV, other climatic variables; RAD, radiation; PRE, precipitation; MOI, moisture

The machine learning model we developed was used to predict ATG cluster diversity on a global scale, leading to the construction of an atlas of the global distribution of ATG diversity at a resolution of 0.167° (Fig. 5a). The findings indicated that densely populated regions, such as India, Eastern China, Southeast Asia, the Middle East, Europe, and certain parts of the United States, exhibited greater diversity of ATG clusters. Similarly, areas with higher temperatures also showed greater diversity in ATG clusters, particularly in certain regions of Africa. Conversely, areas characterized by lower population density and cooler temperatures, such as Siberia, Canada, and the Tibetan Plateau, tended to have lower diversity in the ATG clusters. A similar distribution pattern can be observed in the global prediction atlas of family diversity (Supplementary Fig. S10). However, unlike the diversity patterns, the hotspots of ATG abundance were concentrated mainly in regions with relatively high temperatures, such as Central America and Southeast Asia near the equator. In regions with high population density but relatively lower temperatures than in equatorial regions, such as Eastern China, Europe, and the United States, ATG abundance tended to be lower (Fig. 5b). Despite the differences in ATG diversity and abundance hotspots, their latitudinal trends were similar (Fig. 5c, d), with both showing a decrease from low to high latitudes.

The global distributions of ATG diversity and abundance implied that their main drivers are different. We categorized the variables into eight groups and assessed their relative importance to the prediction model (Supplementary Data 4, 5, 6). We found that for global hotspots of ATG cluster diversity, anthropogenic factors were the most important drivers (23.5%), followed by temperature (19.7%) and soil properties (18.5%) (Fig. 5e). Temperature (27.9%) and anthropogenic factors (25.0%) had similar effects on family diversity (Supplementary Fig. S10). However, for the ATG abundance prediction, temperature was the most important driver (25.4%), and the influence of anthropogenic factors (15.8%) was substantially reduced (Fig. 5f). Thus, anthropogenic factors and temperature were the two most critical drivers for global ATG distribution, but diversity was more affected by anthropogenic factors than abundance. Anthropogenic factors may facilitate microbial exchange across habitats, thereby increasing ATG diversity.

## Discussion

Antagonistic interactions mediated by antimicrobial toxins in microbial communities contribute not only to the composition and relative proportions of microbial members but also to the longer-term stability of a community, playing key roles in ecosystem defense, pathogen invasion, spatial segregation, and diversity [3, 5]. Here, we identified the ubiquity of antimicrobial toxins across global microbial communities, revealing remarkable diversity. Depending on the presence or absence of specific ATG clusters (or families), the composition of ATG clusters (or families) in almost any two microbial communities differed. This suggested that each microbial community may encode a unique set of antimicrobial toxins, resembling a personalized “barcode.” Studies on the human gut microbiota have revealed the collective action of antimicrobial toxins in the community, where strains need to resist attacks from antimicrobial toxins produced by multiple species to survive [69]. Therefore, deciphering the “barcode” of community antimicrobial toxins will enhance our understanding of microbial colonization in different habitats.

Although intercellular transfer of antimicrobial toxins may be a fundamental and widespread characteristic of microorganisms, the known antimicrobial toxins remain limited [5, 7, 10, 35]. The antimicrobial toxin-immunity protein system is also easily confused with toxin-antitoxin systems; however, the toxins encoded by toxin-antitoxin systems are self-toxic agents that are not secreted extracellularly. We previously constructed the first prokaryotic antimicrobial toxin database, which advanced the methodologies for discovering antimicrobial toxins [7, 17, 42, 56]. The antimicrobial toxins analyzed in this study were identified by integrating a series of features from known homologous sequences of antimicrobial toxins. Each identified toxin possesses secretion characteristics and is associated with specific secretion mechanisms, thereby minimizing the risk of false positives. Despite revealing a high diversity of antagonistic weapons in communities, considering that many families remain uncharacterized, we still underestimate the diversity of microbial antimicrobial toxins. Moreover, the presence of ATGs in a community reflects their antagonistic potential and does not necessarily indicate functional expression in the environment. In the future, it will become possible to more comprehensively assess the diversity of antimicrobial toxins and their ecological roles in microbial communities on a global scale as laboratory studies continue to reveal novel antimicrobial toxins and acquire more metatranscriptomic data.

Why have microbial antimicrobial toxin arsenals evolved such remarkable diversity? We found that the vast majority of ATG clusters in habitats are rare, appearing exclusively in specific communities. This phenomenon differs from the distribution pattern of functional gene occupancy, which typically displays bimodality and a right-skewed pattern dominated by widespread types [70, 71]. Instead, similar to the reported taxa distributions in microbial communities, they exhibited a greater left-skewed pattern [72]. This finding indicated that antimicrobial toxins are subject to negative frequency-dependent selection, where rare strategies have advantages. Once resistance to a particular antimicrobial toxin becomes widespread, natural selection favors strains that produce different, rarer toxins, thus maintaining a high diversity of antimicrobial toxins [1]. For ATG abundance, moderate levels were most common; too low an abundance led to insufficient accumulation, whereas too high an abundance resulted in a lack of targets to kill. Most microbial genes exhibited habitat-specific distributions, with only a few found across multiple habitats [73]. However, ATG clusters, while rare, were predominantly distributed across habitats. The sharing of ATGs between habitats occurred mainly because ATG-carrying species can survive in multiple habitats. There are also numerous examples demonstrating that ATGs are part of mobile genetic elements capable of horizontal transfer between genomes and even crossing habitat boundaries [74].

ATG abundance and diversity exhibited widespread habitat variations, yet their distribution patterns and influencing factors differed. Due to the high cost of antimicrobial toxin synthesis, cells require sufficient nutrients to invest in producing these toxins [75]. We found that global ATG abundance hotspots are more influenced by temperature, precipitation, radiation, and moisture than the hotspots of ATG cluster diversity, and these factors are often associated with nutrient availability. The greater abundance of ATGs in host-associated communities than in free-living communities may also be attributed to higher nutrient levels in the former. ATG diversity is positively correlated with biodiversity, with anthropogenic factors influencing global ATG diversity hotspots more than abundance hotspots. Anthropogenic activities have been shown to increase soil antibiotic resistance by introducing microbes carrying antibiotic resistance genes [76]. The ATGs likely followed a similar pattern, with anthropogenic factors enhancing microbial exchange carrying ATGs between habitats, thereby increasing ATG diversity. Notably, we found that ATG diversity in human-associated habitats was significantly greater than that in other animal-associated habitats, with greater ATG sharing between human-associated habitats and natural habitats. These findings suggest that anthropogenic activities are enhancing the enrichment of diverse ATGs in human habitats from other habitats. Given the existing evidence concerning the potential harm of microbial antimicrobial toxins to human health, there may be an increased risk of these toxins inducing diseases or contributing to the development of cancer [5, 22].

Antimicrobial toxins have exhibited significant potential as broad-spectrum bactericides and biotechnological tools [34, 35]. Our study provided baseline information on the global distribution of ATGs for the first time by combining community samples, genome sequences, and environmental constraints. Our work emphasized that microorganisms in nature have evolved a highly diverse array of antimicrobial toxins as antagonistic weapons, providing valuable resources for potential clinical, agricultural, and industrial applications.

## Supplementary Information

Additional file 1: Supplementary Fig. S1. Proportion of genome sequencing in global microbial communities has reached a high level. The proportions of the genome-sequenced cells and taxa in the microbial biomes were evaluated based on the alignments between the 262,011 ASV sequence data and 217,614 sequenced genome information. The results showed that the median proportions of genome-sequenced cells and taxa reached 40.2% (20.5%-88.1%) and 21.3% (11.4%-57.0%), respectively, at 100% identity in the 16S-V4 region for the 10,000 analyzed samples. For the box plots, the middle line represents the median, the box indicates the 25th-75th percentiles, and the error bars represent the 10th-90th percentiles of the observations.

Additional file 2: Supplementary Fig. S2. Positive correlations between the number of ATG clusters and biodiversity in the community. Statistics based on 10,000 EMP samples. Alpha diversity indices included the Chao1 index, Faith's PD index, and Shannon index. Green dots represent samples from free-living habitats, while red dots represent samples from host-associated habitats. The gray line indicates the best linear fit, with the shaded area representing the 95% confidence interval and *r*^*2*^ representing the coefficient of determination.

Additional file 3: Supplementary Fig. S3. Differences in ATG family diversity among different habitats. Red represents host-associated habitats, green represents free-living habitats, and yellow represents human-associated habitats. For the boxplot, the middle line represents the median, the box represents the 25th-75th percentiles, and the error bars represent the 10th-90th percentiles of the observations. Comparisons between bins were conducted using the Wilcoxon rank-sum test, *** *P* < 0.001.

Additional file 4: Supplementary Fig. S4. Impact of community environmental factors on ATG abundance and diversity. A total of 2,381 samples with recorded temperature information, 1,183 samples with recorded pH values, and 597 samples with recorded salinity information were analyzed. The hypersaline samples are defined by a salinity of 50 psu. The green dots represent samples from free-living habitats, whereas the red dots represent samples from host-associated habitats.

Additional file 5: Supplementary Fig. S5. Abundance distribution of the ATG cluster in 16 habitats. The gray line indicates the best Gaussian model fit, with *R*^*2*^ representing the coefficient of determination. Red indicates host-associated habitats, whereas green indicates free-living habitats.

Additional file 6: Supplementary Fig. S6. Relationships between ATG cluster occupancy and abundance in 16 habitats. The gray line indicates the best linear fit, with the shaded area representing the 95% confidence interval and *r*^*2*^ representing the coefficient of determination. Red indicates host-associated habitats, whereas green indicates free-living habitats.

Additional file 7: Supplementary Fig. S7. Feature selection and hyperparameter tuning for the random forest algorithm. ATG abundance and diversity were predicted based on tenfold cross-validation. A recursive feature elimination algorithm was employed to select the optimal feature set for prediction, and a grid search was used to select the optimal hyperparameter combination.

Additional file 8: Supplementary Fig. S8. The coefficient of variation was used as a measure of prediction accuracy to predict the uncertainty map of ATG abundance and diversity.

Additional file 9: Supplementary Fig. S9. Relationships between predicted and observed ATG abundance or diversity in the training and testing sets. The observed results were obtained through analysis of EMP samples, whereas the predicted results were generated using a random forest model.

Additional file 10: Supplementary Fig. S10. Global biogeographic patterns of ATG family diversity. a. Global distribution maps of ATG family diversity. Global ATG family diversity was predicted based on a random forest model using 97 spatial covariates. Four-fifths of the samples were used as the training set, whereas one-fifth served as the testing set (for family diversity, the training set tenfold cross-validation *R*^*2*^ = 0.679, and the testing set *R*^*2*^ = 0.572; Supplementary Fig. S9). b. Latitudinal distribution of global ATG family diversity. The dashed lines represent the average of global ATG family diversity. c. Relative importance of each category of variables for predicting ATG family diversity. ANT: Anthropogenic, TEM: Temperature, SP: Soil properties, LUAO: Land use and others, OCV: Other climatic variables, RAD: Radiation, PRE: Precipitation, MOI: Moisture.

Additional file 11: Supplementary Data 1. Information on the identified ATGs in microbial communities.

Additional file 12: Supplementary Data 2. Distribution data of ATGs in microbial communities.

Additional file 13: Supplementary Data 3. Spatial covariates used in machine learning.

Additional file 14: Supplementary Data 4. Relative importance of variables for predicting ATG abundance.

Additional file 15: Supplementary Data 5. Relative importance of variables for predicting ATG cluster diversity.

Additional file 16: Supplementary Data 6. Relative importance of variables for predicting ATG family diversity.

## Data Availability

Data is provided within the manuscript or supplementary information files.

## Abbreviations

ATGs: Antimicrobial toxin genes
eCISs: Extracellular contractile injection systems
T6SS: Type VI secretion system
EMP: Earth Microbiome Project
ASVs: Amplicon sequence variants
EMPO: EMP ontology
LDG: Latitudinal diversity gradient
