# Supplementary figures and images for: Global biogeography and projection of antimicrobial toxin genes
Source: Microbiome. 2025 Feb 4;13:40. doi: 10.1186/s40168-025-02038-5 (PMC11796102; doi:10.1186/s40168-025-02038-5)

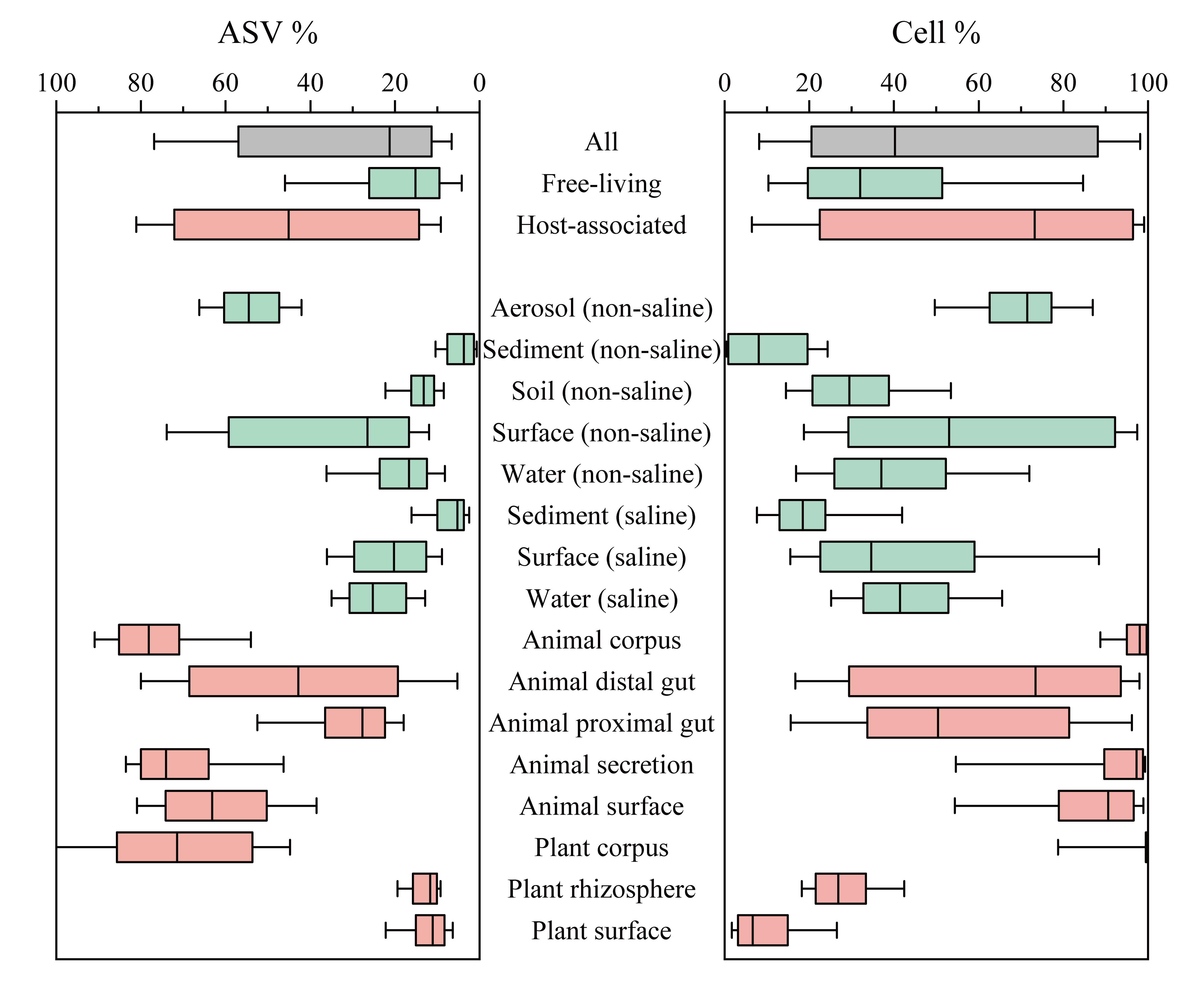

Supplement: Supplementary file 2 — Additional file 1: Supplementary Fig. S1. Proportion of genome sequencing in global microbial communities has reached a high level. The proportions of the genome-sequenced cells and taxa in the microbial biomes were evaluated based on the alignments between the 262,011 ASV sequence data and 217,614 sequenced genome information. The results showed that the median proportions of genome-sequenced cells and taxa reached 40.2% (20.5%-88.1%) and 21.3% (11.4%-57.0%), respectively, at 100% identity in the 16S-V4 region for the 10,000 analyzed samples. For the box plots, the middle line represents the median, the box indicates the 25th-75th percentiles, and the error bars represent the 10th-90th percentiles of the observations. [file 40168_2025_2038_MOESM1_ESM.tif]

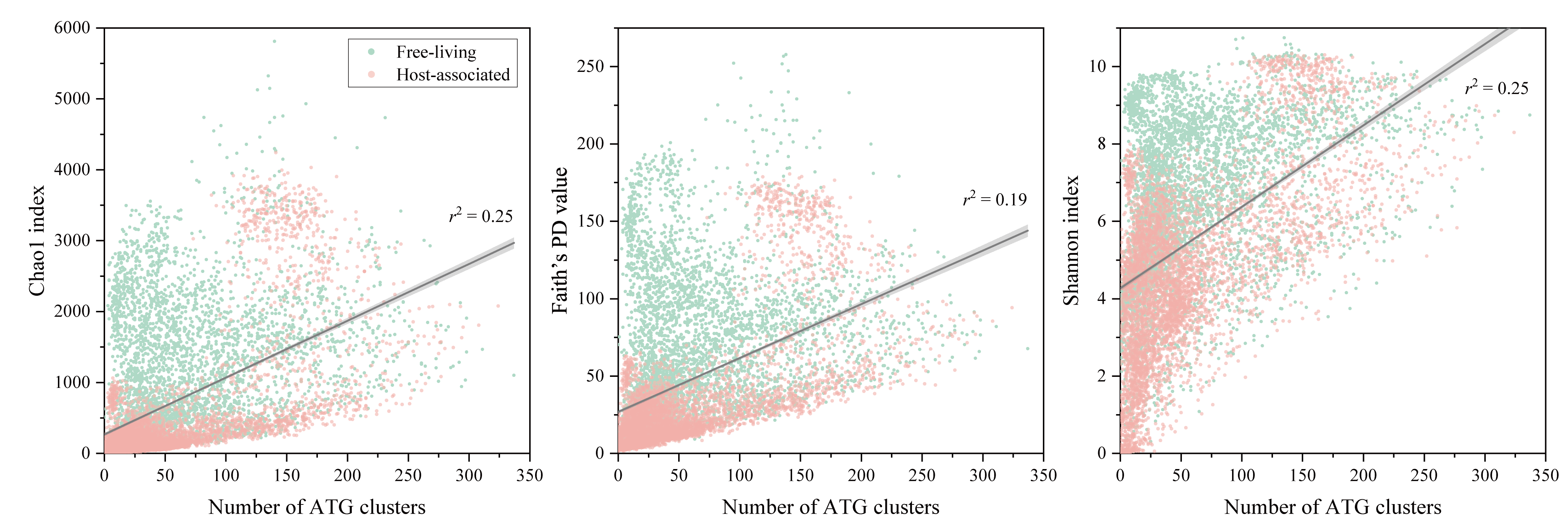

Supplement: Supplementary file 3 — Additional file 2: Supplementary Fig. S2. Positive correlations between the number of ATG clusters and biodiversity in the community. Statistics based on 10,000 EMP samples. Alpha diversity indices included the Chao1 index, Faith's PD index, and Shannon index. Green dots represent samples from free-living habitats, while red dots represent samples from host-associated habitats. The gray line indicates the best linear fit, with the shaded area representing the 95% confidence interval and r2 representing the coefficient of determination. [file 40168_2025_2038_MOESM2_ESM.tif]

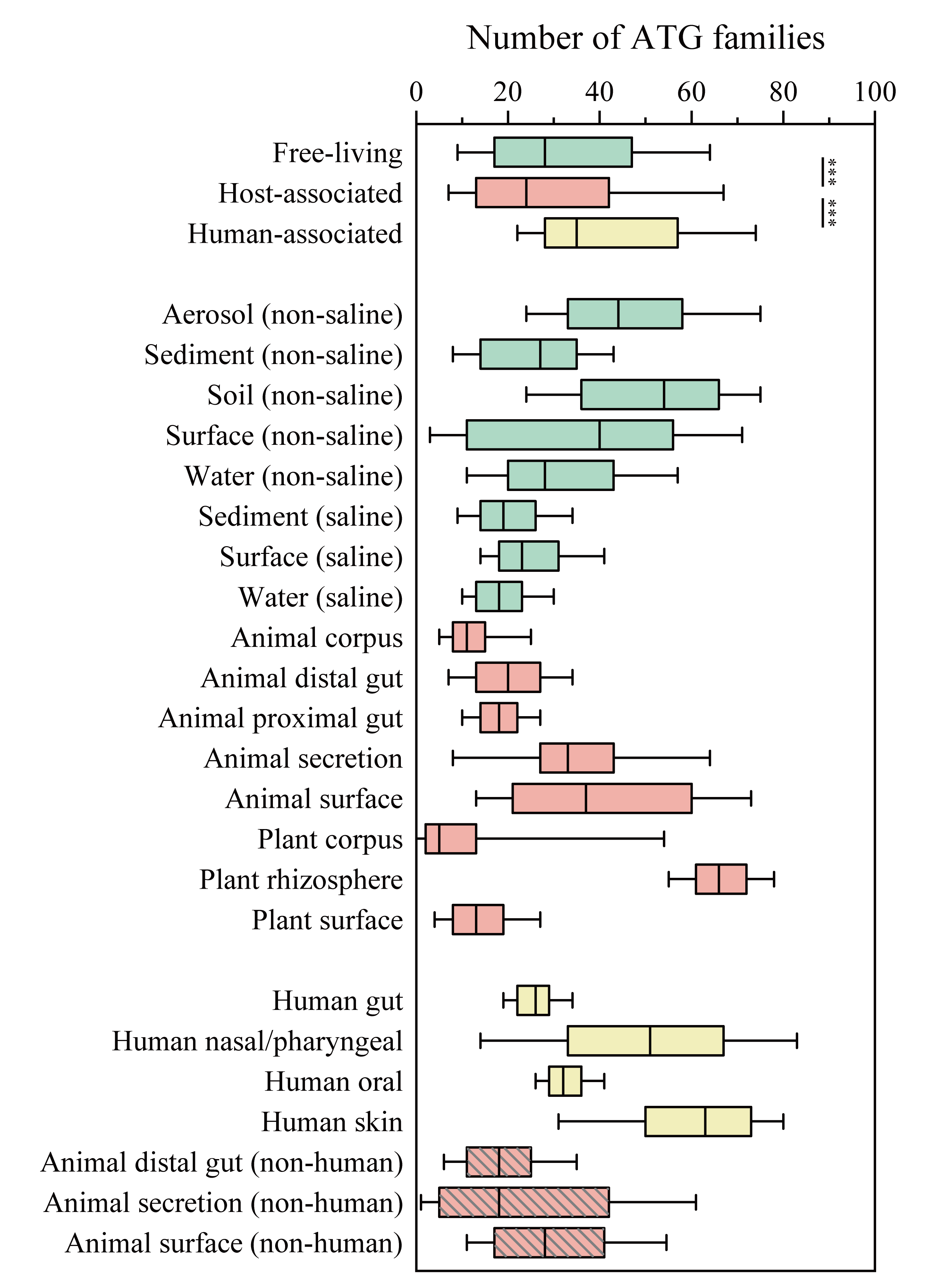

Supplement: Supplementary file 4 — Additional file 3: Supplementary Fig. S3. Differences in ATG family diversity among different habitats. Red represents host-associated habitats, green represents free-living habitats, and yellow represents human-associated habitats. For the boxplot, the middle line represents the median, the box represents the 25th-75th percentiles, and the error bars represent the 10th-90th percentiles of the observations. Comparisons between bins were conducted using the Wilcoxon rank-sum test, *** P < 0.001. [file 40168_2025_2038_MOESM3_ESM.tif]

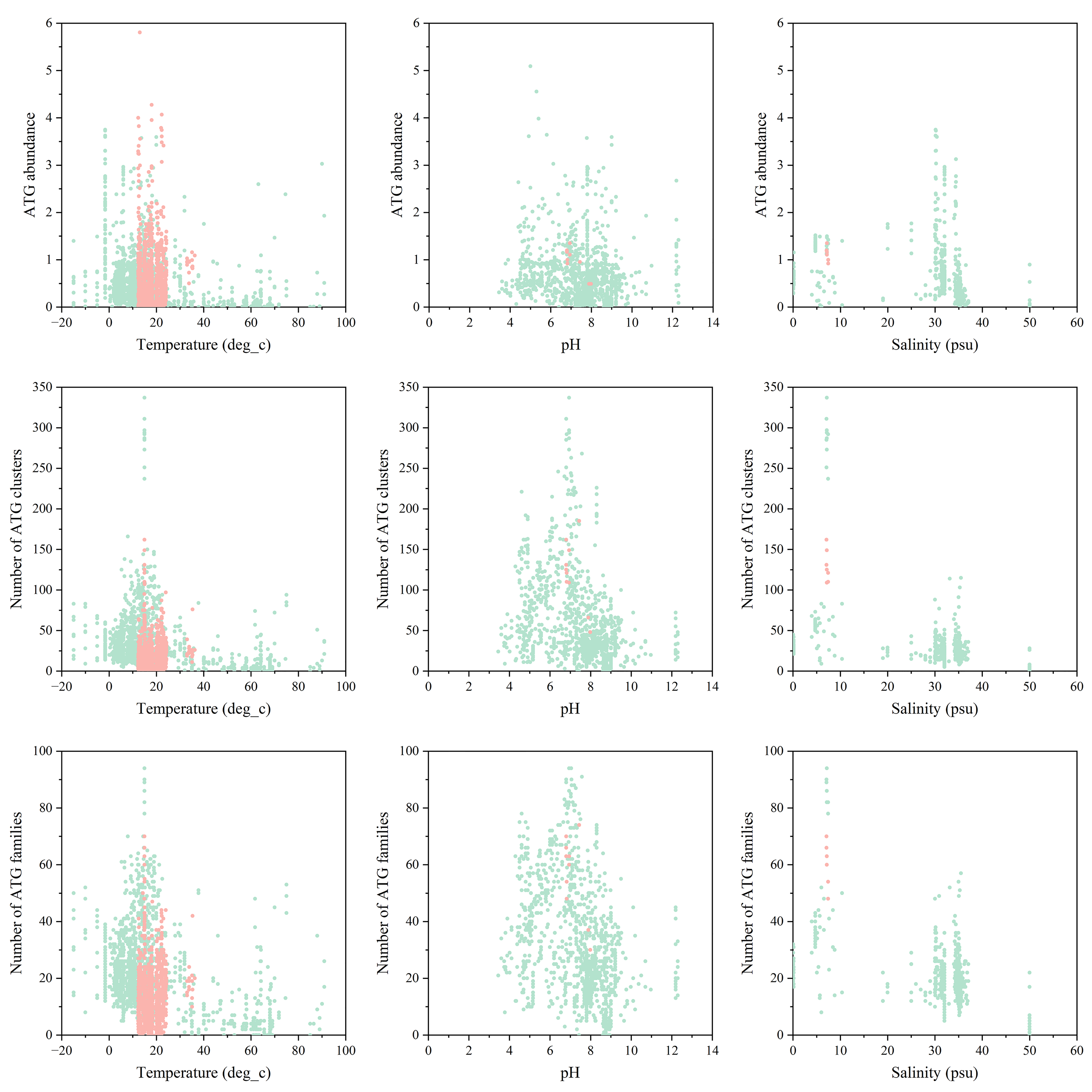

Supplement: Supplementary file 5 — Additional file 4: Supplementary Fig. S4. Impact of community environmental factors on ATG abundance and diversity. A total of 2,381 samples with recorded temperature information, 1,183 samples with recorded pH values, and 597 samples with recorded salinity information were analyzed. The hypersaline samples are defined by a salinity of 50 psu. The green dots represent samples from free-living habitats, whereas the red dots represent samples from host-associated habitats. [file 40168_2025_2038_MOESM4_ESM.tif]

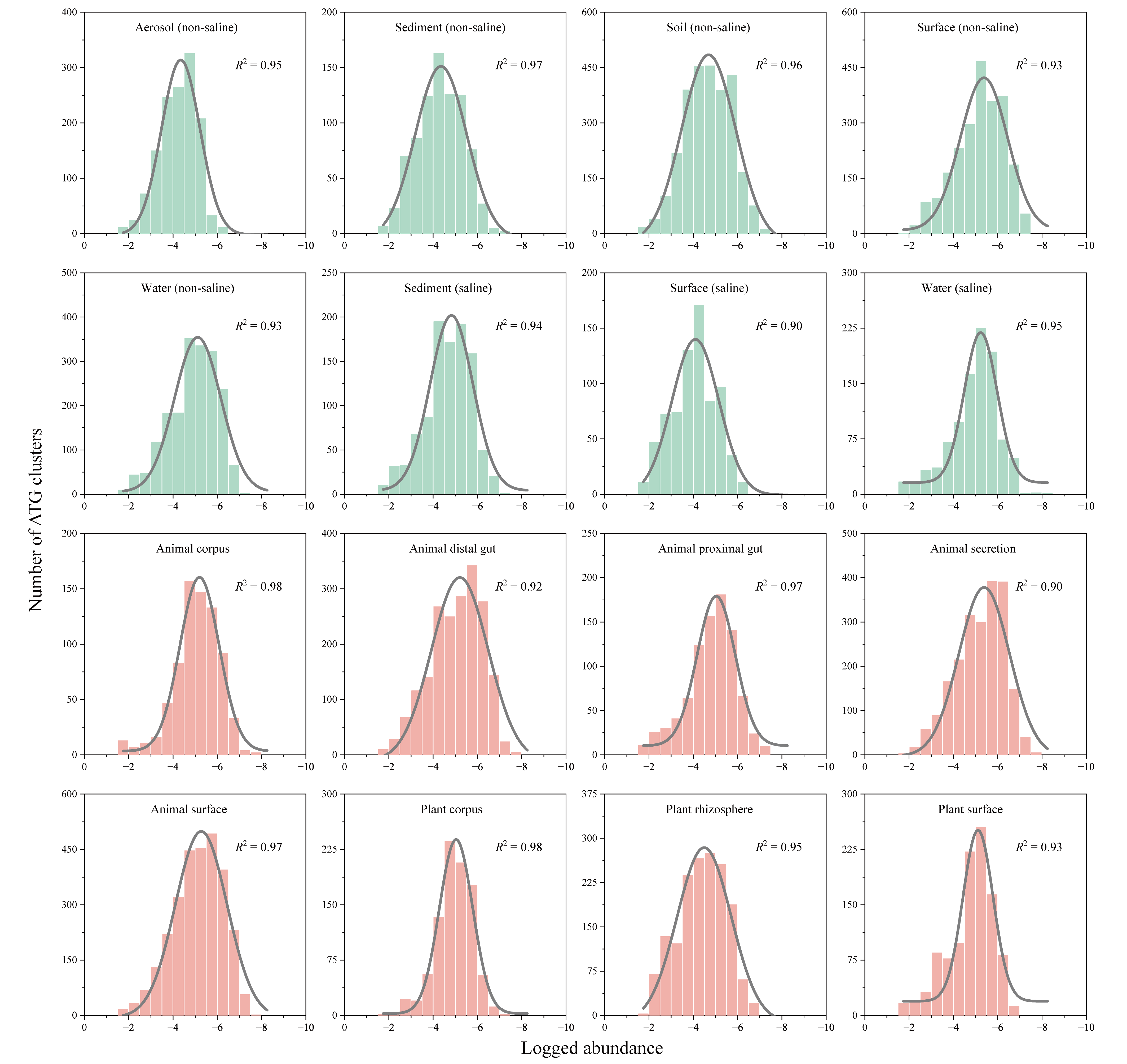

Supplement: Supplementary file 6 — Additional file 5: Supplementary Fig. S5. Abundance distribution of the ATG cluster in 16 habitats. The gray line indicates the best Gaussian model fit, with R2 representing the coefficient of determination. Red indicates host-associated habitats, whereas green indicates free-living habitats. [file 40168_2025_2038_MOESM5_ESM.tif]

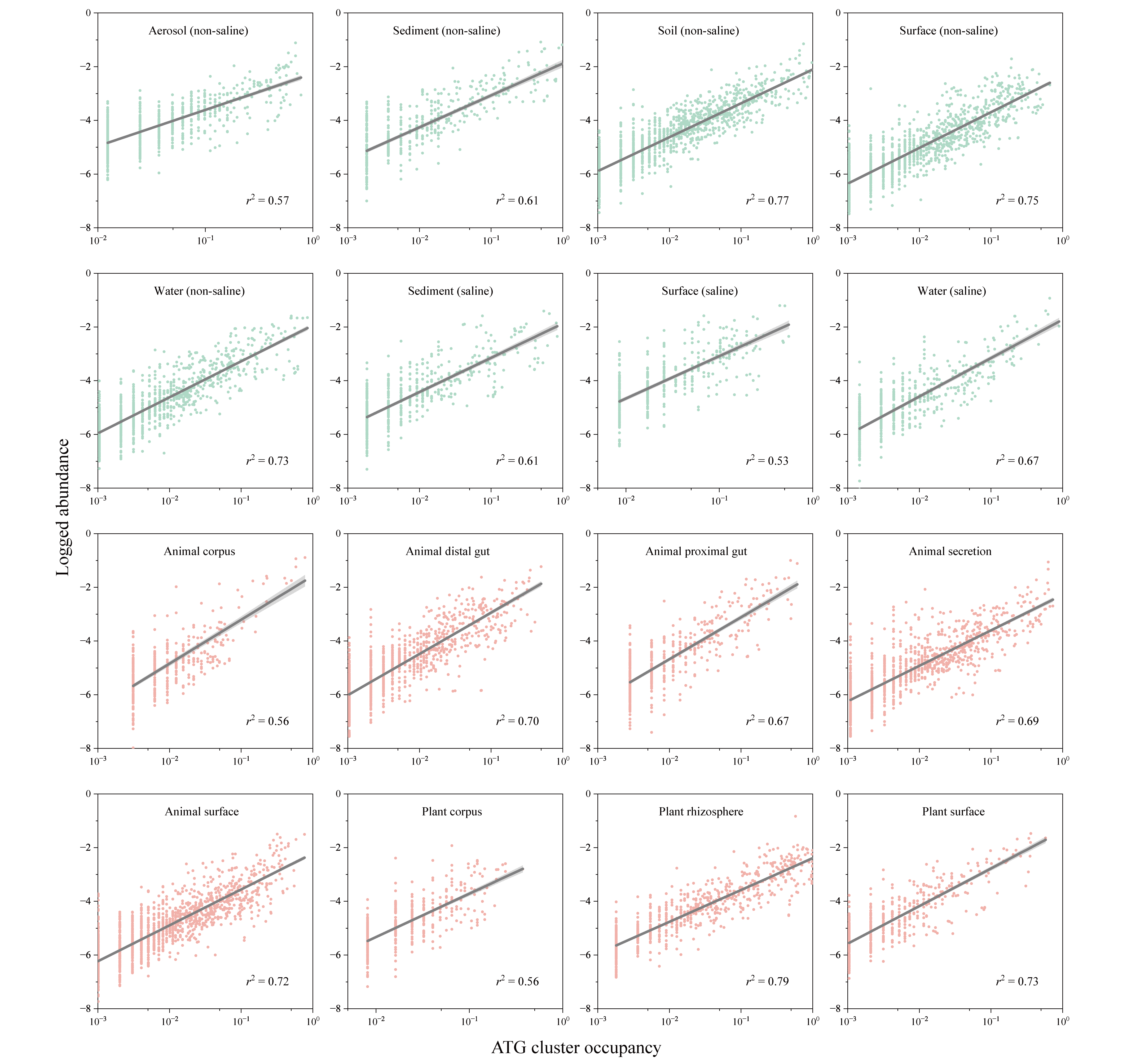

Supplement: Supplementary file 7 — Additional file 6: Supplementary Fig. S6. Relationships between ATG cluster occupancy and abundance in 16 habitats. The gray line indicates the best linear fit, with the shaded area representing the 95% confidence interval and r2 representing the coefficient of determination. Red indicates host-associated habitats, whereas green indicates free-living habitats. [file 40168_2025_2038_MOESM6_ESM.tif]

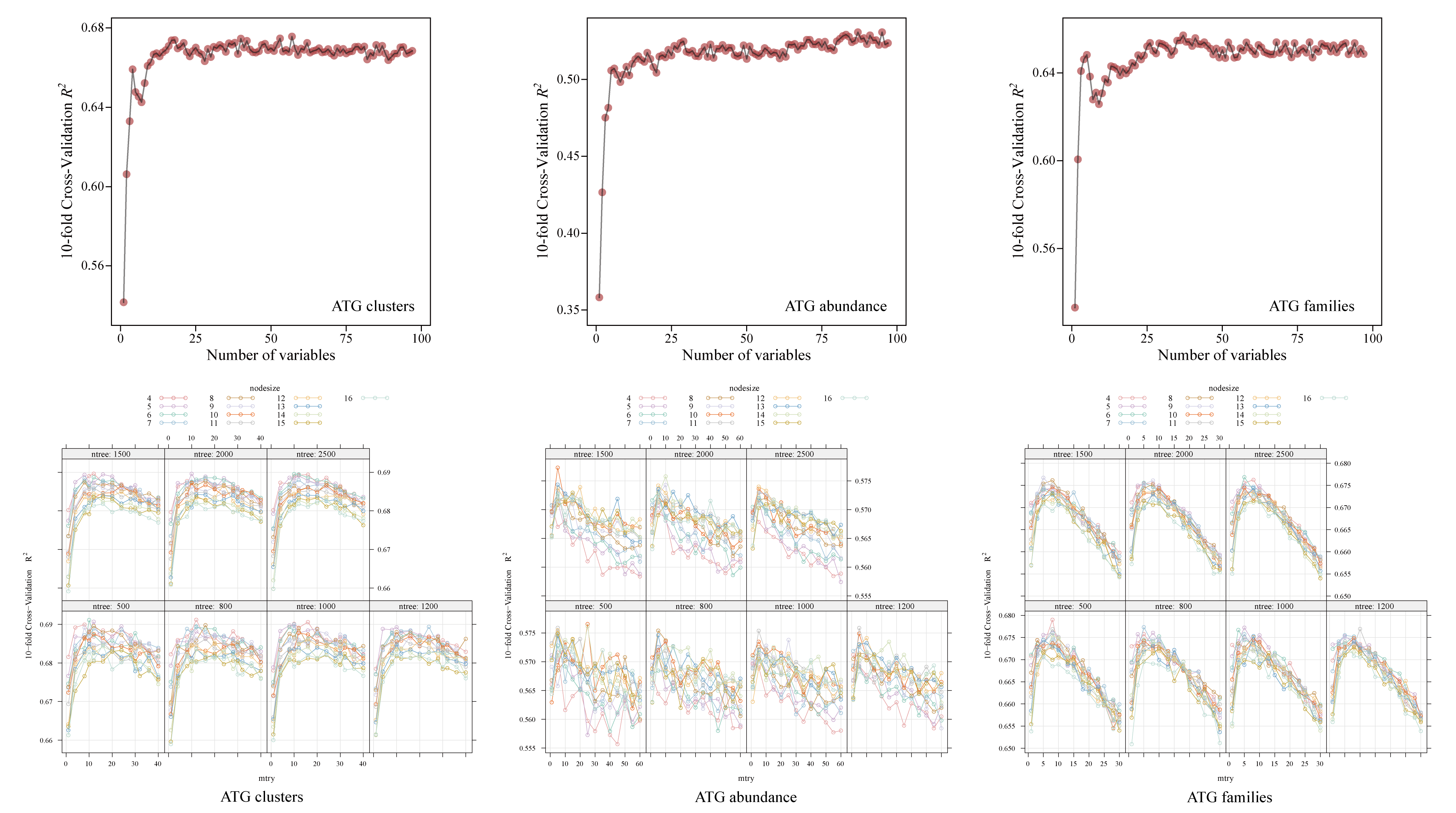

Supplement: Supplementary file 8 — Additional file 7: Supplementary Fig. S7. Feature selection and hyperparameter tuning for the random forest algorithm. ATG abundance and diversity were predicted based on tenfold cross-validation. A recursive feature elimination algorithm was employed to select the optimal feature set for prediction, and a grid search was used to select the optimal hyperparameter combination. [file 40168_2025_2038_MOESM7_ESM.tif]

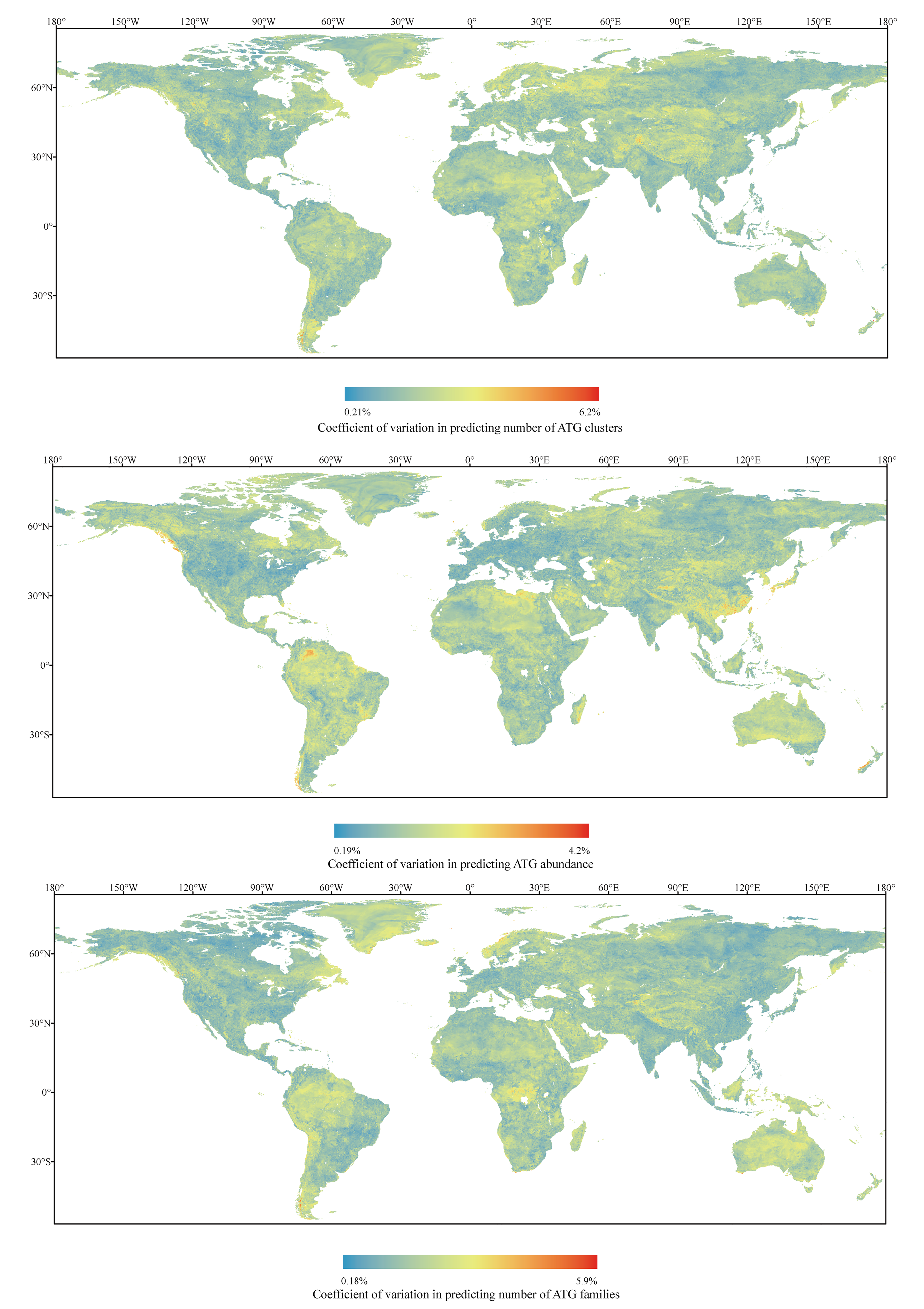

Supplement: Supplementary file 9 — Additional file 8: Supplementary Fig. S8. The coefficient of variation was used as a measure of prediction accuracy to predict the uncertainty map of ATG abundance and diversity. [file 40168_2025_2038_MOESM8_ESM.tif]

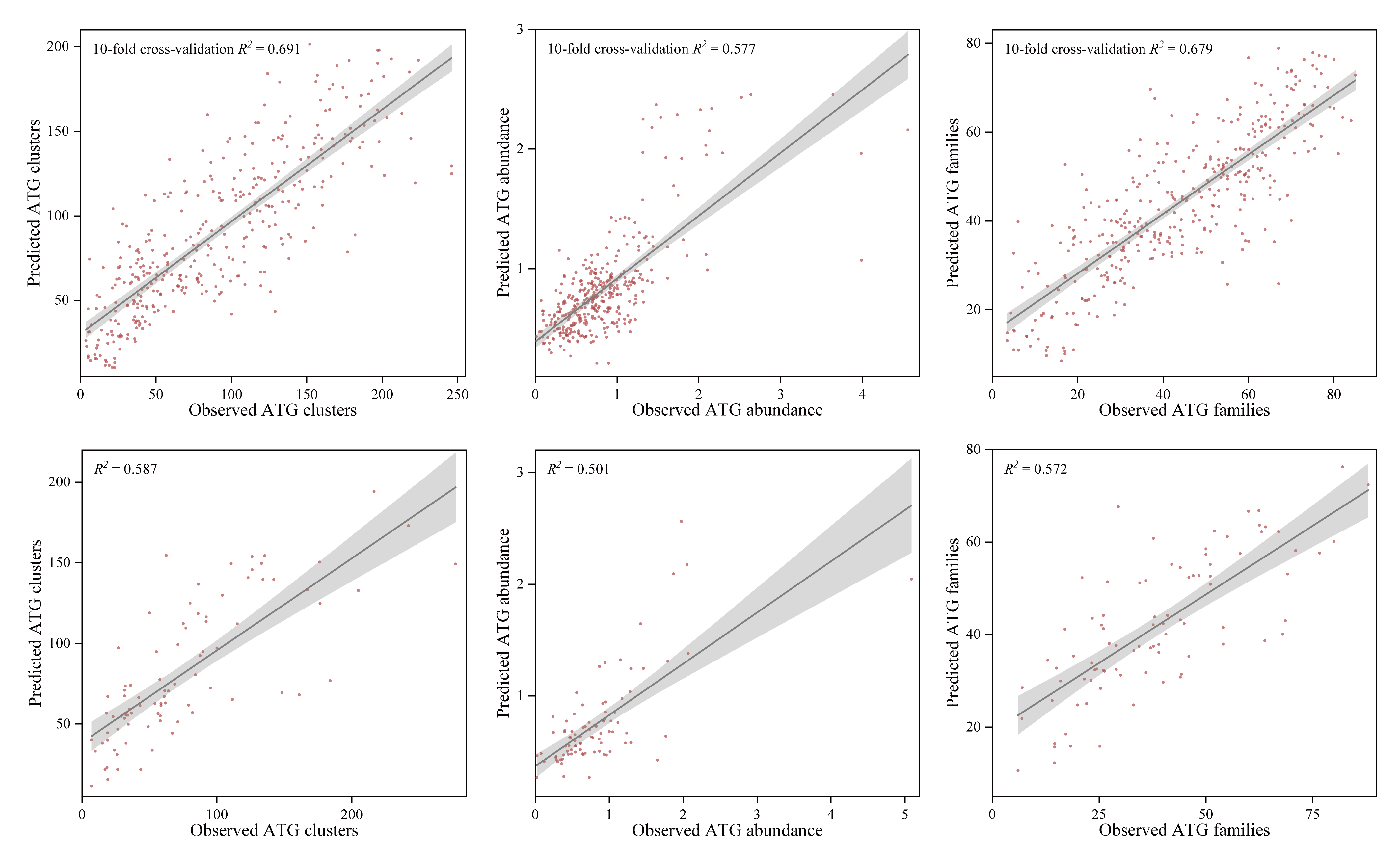

Supplement: Supplementary file 10 — Additional file 9: Supplementary Fig. S9. Relationships between predicted and observed ATG abundance or diversity in the training and testing sets. The observed results were obtained through analysis of EMP samples, whereas the predicted results were generated using a random forest model. [file 40168_2025_2038_MOESM9_ESM.tif]

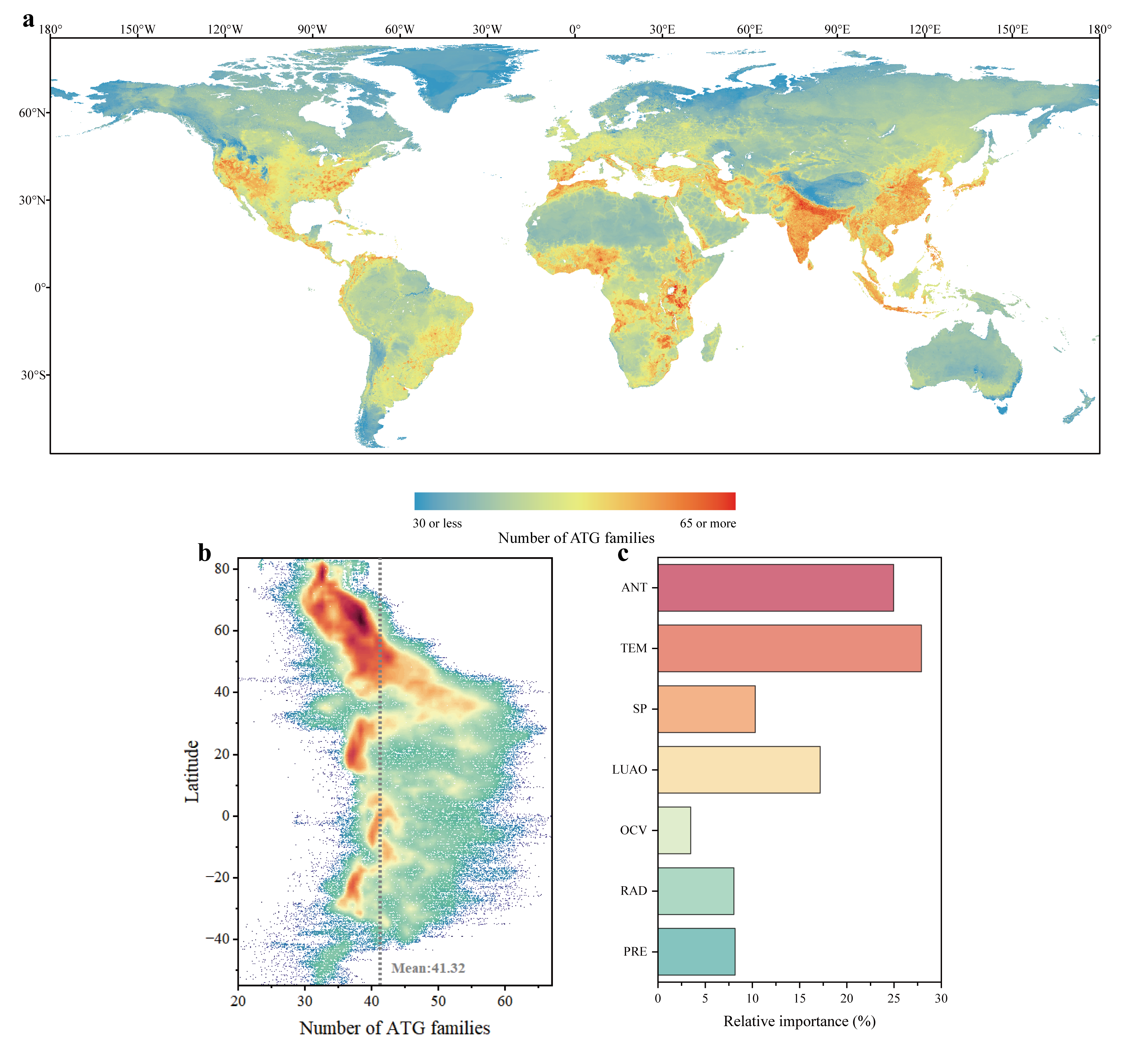

Supplement: Supplementary file 11 — Additional file 10: Supplementary Fig. S10. Global biogeographic patterns of ATG family diversity. a. Global distribution maps of ATG family diversity. Global ATG family diversity was predicted based on a random forest model using 97 spatial covariates. Four-fifths of the samples were used as the training set, whereas one-fifth served as the testing set (for family diversity, the training set tenfold cross-validation R2 = 0.679, and the testing set R2 = 0.572; Supplementary Fig. S9). b. Latitudinal distribution of global ATG family diversity. The dashed lines represent the average of global ATG family diversity. c. Relative importance of each category of variables for predicting ATG family diversity. ANT: Anthropogenic, TEM: Temperature, SP: Soil properties, LUAO: Land use and others, OCV: Other climatic variables, RAD: Radiation, PRE: Precipitation, MOI: Moisture. [file 40168_2025_2038_MOESM10_ESM.tif]
